# Monounsaturated fatty acids protect against palmitate-induced lipoapoptosis in human umbilical vein endothelial cells

**DOI:** 10.1371/journal.pone.0226940

**Published:** 2019-12-31

**Authors:** Dustin M. Lee, Kyle J. Sevits, Micah L. Battson, Yuren Wei, Kimberly A. Cox-York, Christopher L. Gentile

**Affiliations:** Department of Food Science & Human Nutrition, Colorado State University, Fort Collins, CO, United States of America; Max Delbrueck Center for Molecular Medicine, GERMANY

## Abstract

Diets high in saturated fatty acids are linked to increased cardiovascular disease risk, whereas monounsaturated fatty acids have been associated with improved cardiovascular outcomes. Accordingly, cell culture studies have demonstrated that saturated fatty acids, particularly long chain saturated fatty acids such as palmitate, induce dysfunction and cell death in a variety of cell types, and monounsaturated fatty acids may confer protection against palmitate-mediated damage. The aim of the present study was to examine whether monounsaturated fatty acids could protect against palmitate-mediated cell death in endothelial cells, to determine if AMPK inactivation and activation (via compound C and AICAR, respectively) underlies both palmitate-induced damage and monounsaturated fatty acid-mediated protection, and to explore the role of ER stress in this context. Human umbilical vein endothelial cells were examined for cell viability and apoptosis following treatment for 24 hours with palmitate (0.25 and 0.5mM) alone or in combination with the monounsaturated fatty acids oleate or palmitoleate (0.25 and 0.5mM), AICAR, compound C, 4μ8C, or TUDCA. Compared to control cells, palmitate significantly decreased cell viability and increased apoptosis in a dose-dependent manner. The monounsaturated fatty acids oleate and palmitoleate completely prevented the cytotoxic effects of palmitate. Although palmitate induced markers of ER stress, chemical inhibition of ER stress did not prevent palmitate-induced lipoapoptosis. Conversely, the AMPK activator AICAR (0.1 and 0.5mM) conferred protection from palmitate mediated-alterations in viability, apoptosis and ER stress, whereas the AMPK inhibitor compound C (20 and 40μM) significantly exacerbated palmitate-mediated damage. Lastly, co-incubation with palmitate, monounsaturated fatty acids, and compound C significantly mitigated the protective effects of both oleate and palmitoleate. In conclusion, monounsaturated fatty acids confer protection against the cytotoxic effects of palmitate in vascular endothelial cells; and palmitate-mediated damage, as well as monounsaturated-mediated protection, are due in part to inactivation and activation, respectively, of the metabolic regulator AMPK. These results may have implications for understanding the deleterious effects of high saturated fat diets on cardiovascular dysfunction and disease risk.

## Introduction

The vascular endothelium is comprised of a single-cell monolayer that lines the internal surface of blood vessels and thus serves as the primary interface between luminal blood and underlying tissues. Given this strategic location, endothelial cells are capable of detecting mechanical and chemical changes within the luminal environment and orchestrating autocrine and paracrine responses that help regulate cardiovascular function. Thus, integrity of the endothelial cell lining is critical to maintaining overall cardiovascular homeostasis, and endothelial cell dysfunction has been implicated in the pathogenesis of various cardiovascular abnormalities and is predictive of future cardiovascular events [1, 2].

Several studies have demonstrated that endothelial cell apoptosis is an important underlying cause of endothelial dysfunction [3, 4]. Apoptosis compromises the endothelial cell barrier and alters the balance of endothelium-derived substances towards a pro-inflammatory, pro-thrombotic and oxidative phenotype [3]. Given their location, endothelial cells are exposed to numerous luminal substances that can prevent or promote apoptosis. Among these substances, circulating fatty acids derived from the diet or from triacylglycerol hydrolysis are potent stimulators of cell death pathways. In healthy individuals, free fatty acids circulate at approximately 400 μmol/L, but can increase above 600 μmol/L in metabolic disease states such as obesity or type 2 diabetes [5, 6]. Elevated circulating fatty acids impair endothelium-dependent dilation [7, 8], and lipid-mediated endothelial cell apoptosis (or lipoapoptosis) has been suggested as an important mechanism linking elevated circulating fatty acids with cardiovascular disease [9].

Several endothelial cell modifications occur during the development of endothelial dysfunction, including endothelial cell activation and induction of endoplasmic reticulum (ER) stress. Endothelial cell activation is characterized by the expression of adhesion molecules (i.e. selectins or ICAM-1) which promote inflammation within the vessel wall [10]. Dysfunction within the ER, broadly termed ER stress, induces the unfolded protein response (UPR). While the UPR is critical in restoring ER homeostasis, chronic activation of this process has been implicated in the pathophysiology of metabolic diseases [11] including the development of endothelial dysfunction [12]. Mitigating cellular adhesion or ER stress has been shown to alleviate several cardiometabolic diseases [13, 14]. Additionally, we have previously shown that chemical inhibition of ER stress reduces arterial stiffness and improves endothelial dysfunction in type 2 diabetic mice [15].

The cellular effects of fatty acids vary depending on the chain length and saturation state. For example, high dietary intake of long chain saturated fatty acids such as palmitate (C16:0) and stearate (C18:0) is associated with elevated risk of CVD and diabetes [16, 17]. Palmitate, the most prevalent saturated fatty acid in circulation [17], also impairs endothelium-dependent vasodilation [7] and is a potent stimulator of endothelial cell lipoapoptosis [18]. Conversely, diets high in mono- and polyunsaturated fatty acids are generally cardioprotective [16]. Interestingly, the addition of unsaturated fatty acids to hepatocytes [19, 20] and pancreatic β-cells [21] has been shown to protect against saturated fatty acid-mediated lipoapoptosis. In endothelial cells, the monounsaturated fatty acid oleate prevents palmitate- and stearate-induced cell toxicity [22, 23] although the effects on apoptosis are unknown. Furthermore, the mechanisms by which monounsaturated fatty acids protect against saturated fatty acids in endothelial cells are unclear. With this background, the goal of the present study was to examine the effects of the saturated fatty acid palmitate, with and without monounsaturated fatty acids, on endothelial cell viability and apoptosis; and to begin to explore potential mechanisms (i.e. ER stress) underlying the hypothesized deleterious and protective effects of saturated and monounsaturated fatty acids, respectively.

## Methods

### Experimental protocol

Human umbilical vein endothelial cells (HUVEC) from pooled donors were purchased at passage zero (Lonza, Basel, Switzerland; #CC-2519) and used for experiments in passages 2–5. HUVECs proliferated on 10cm collagen coated plastic cell culture plates to 100% confluence in EGM-2 media containing 2% fetal bovine serum (FBS) (Lonza, Basel, Switzerland; #CC-3162 & CC-4176) in humidified chambers at 37°C, 5% CO_2_. Once cells reached confluence, they were transferred to 96-well collagen coated plates for treatments and subsequent experiments.

### Fatty acid and other treatments

The saturated fatty acid palmitate, and monounsaturated fatty acids oleate and palmitoleate were used in the present study. Fatty acid stock solutions of palmitate (PA), oleate (Ole), and palmitoleate (PO) (Sigma, St. Louis, MO, USA; #PO500, O1008, 76169) (250mM) were first prepared by dilution into 200 proof ethanol. Next, a 10% FFA-free Bovine Serum Albumin (BSA) stock (weight/volume) was made by dilution into EBM-2 media with 0% FBS (Lonza, Basel, Switzerland; #CC-3156). Finally, 5mM fatty acid solutions were made by dilution into the 10% FFA-free BSA at a 2:1 molar ratio and given 1 hour to complex at 37°C. Final treatment dilutions of 0.5 and 0.25mM were made by dilution into EGM-2 without FBS. HUVECs were serum starved in EGM-2, 0% FBS for 4 hours prior to treatments. Compound C (Sigma-Aldrich, St. Louis, MO, USA; #P5499) and 5-aminoimidazole-4-carboxamine ribonucleotide (AICAR) (Sigma-Aldrich, St. Louis, MO, USA; #A9978) were dissolved in DMSO and applied to cells 1 and 2 hours, respectively, prior to fatty acid treatments. Tauroursodeoxycholic acid (TUDCA) (MilliporeSigma, #580549) was dissolved in EGM-2 medium and applied to cells 2 hours prior to fatty acid treatments. The IRE1 inhibitor, 7-Hydroxy-4-methyl-2-oxo-2*H*-1-benzopyran-8-carboxaldehyde (4μ8C) (TOCRIS Bioscience, #4479) was dissolved in DMSO and co-treated with fatty acid treatments. After serum starvation and pretreatments, fatty acid treatments were applied and cells remained in the incubator for 24 hours. HUVECs were then assessed for viability, apoptosis, or gene expression.

**Cell viability** was assessed by measuring ATP using CellTiter-Glo luminescent assay (Promega, Madison, WI, USA; #G9242). This assay determines the number of viable cells by quantifying ATP and thus indicating the presence of metabolically active cells. In short, luciferase catalyzes the reaction of luciferin and ATP leading to a fluorescent light emission, which is quantified by luminescent intensity.

**Cell apoptosis** was assessed by measuring cleavage into a luciferase substrate via activated caspase-3 or -7 using Caspase-Glo 3/7 luminescent assay (Promega, Madison, WI; #G8091). This assay measures caspase-3 and -7 activity, which are involved in apoptotic cell death. Briefly, a portion of caspase is cleaved, which acts as a substrate for luciferase and results in a fluorescent light emission quantified by luminescent intensity.

### RNA isolation and analyses

Cells were washed with phosphate-buffered saline (PBS) and total RNA from cells was extracted using TRIzol reagent (Invitrogen, Carlsbad, CA; #15596026) according to the manufacturer’s instructions. For Real Time PCR, samples were run in duplicate using a Bio-Rad CFX Connect Real-Time PCR Detection System. PCR efficiency for all primers was between 90% and 105% and linear over 5 orders of magnitude. Target genes (ICAM-1, E-selectin, sXBP1, ATF4, ATF5, CHOP, GADD34, GRP78) were normalized to the constitutively expressed genes β2-microglobulin (β2M) or glyceraldehyde 3-phosphate dehydrogenase (GAPDH). Primer sequences are shown in Table 1. Data were normalized by calculating the ΔCq for each sample, which was derived by subtracting the Cq of the reference gene of interest. Relative quantitation (ΔΔCq) was derived by subtracting the ΔCq for the experimental sample from the average ΔCq of the control group. Fold change differences were calculated by 2^ΔΔCq^ as shown in data figures.

**Table 1:** Sequence of RT-qPCR primers.

Target Gene	Sequence
β2M	(s) CAGCGTACTCCAAAGATTCAGG (as) AGTCAACTTCAATGTCGGATGG
GAPDH	(s) TCTATAAATTGAGCCCGCAGC (as) CGCCCAATACGACCAAATCC
ICAM-1	(s) GTCCTGTATGGCCCCCGACT (as) GGGCAGTGGGAAAGTGCCAT
E-selectin	(s) AGGTTCCTTCCTGCCAAGTGGT (as) TGTCCGAGCTGCAGAGCCAT
sXBP1	(s) GGCATTCTGGACAAGTTGG (as) TAGGCAGGAAGATGGCTTTGG
ATF4	(s) CATCTGTATGAGCCCTGAGTC (as) CGAGAACCACGAGGAACACC
ATF5	(s) AGGCTGGATCCTCAAAATCAC (as) CGGCGACACTCTTCCCTCTG
CHOP	(s) CACTCTTGACCCTGCTTCTC (as) TCTGACTGGAATCTGGAGAG
GADD34	(s) GAAGAGGGAGTTGCTGAAGAGG (as) GGAGACAAGGCAGAAGTAGAGG
GRP78	(s) GAACGTCTGATTGGCGATGC (as) TCAACCACCTTGAACGGCAA

β2M, β2-microglobulin; GAPDH, glyceraldehyde 3-phosphate dehydrogenase; ICAM-1, intracellular adhesion molecule 1; sXBP1, X-box binding protein 1; ATF4, activating transcription factor 4; ATF5, activating transcription factor 5; CHOP, C/EBP homologous protein; GADD34, growth arrest and DNA-damage inducible-34; GRP78, glucose-regulated protein-78.

### Statistical analysis

Statistical comparisons were made using one-way ANOVAs with Tukey’s post hoc test (SPSS for Windows, release 25.0.0.1; SPSS. Chicago, IL, USA). The level of significance was set at p<0.05. Data are reported as mean ± standard error of mean (SEM) for independent experiments (sample size), with each experiment including at least 2 technical replicates.

## Results

**Fig 1:**
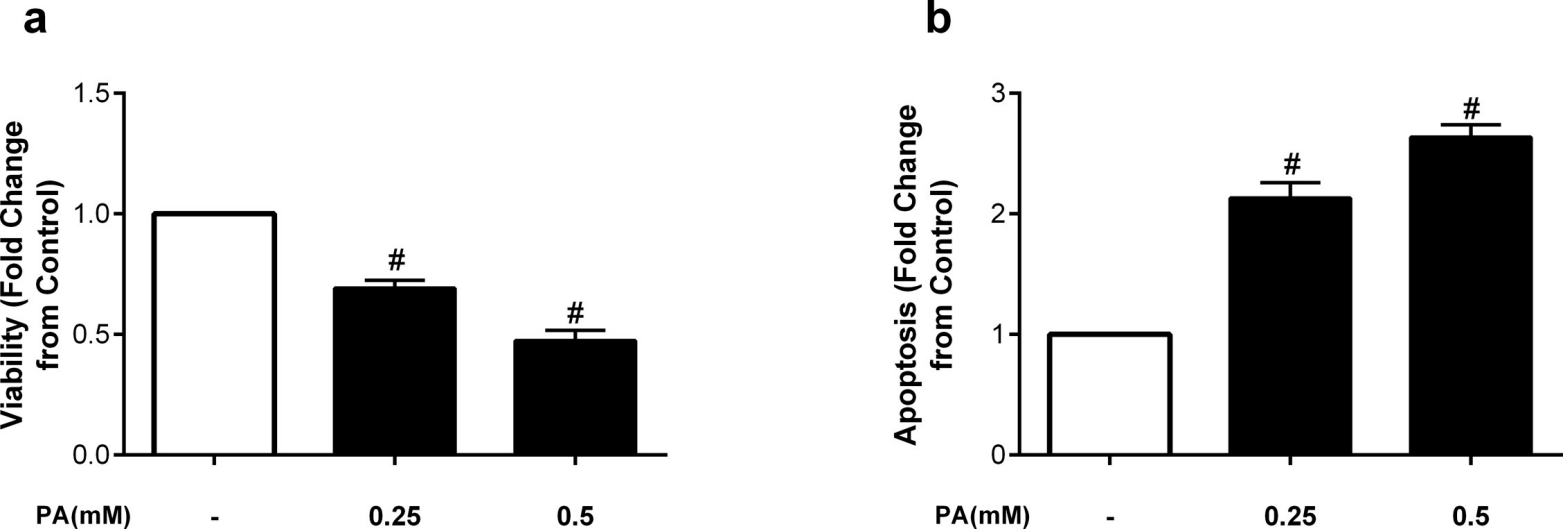
The saturated fatty acid palmitate decreases viability and induces apoptosis in HUVECs. Fold change from control in a) viability and b) apoptosis after treatment with palmitate (PA) at 0.25 or 0.5mM. Data are expressed as mean±SEM; n = 7–8 independent experiments; #p<0.05 vs all.

We first examined whether the saturated fatty acid palmitate altered endothelial cell viability and apoptosis. Endothelial cells were incubated for 24h with 0.25mM and 0.5mM of palmitate. As shown in Fig 1, endothelial cell viability was significantly reduced following incubation with 0.25mM palmitate, and further reduced with 0.5mM, such that viability was approximately half the control condition at the higher concentration of palmitate (Fig 1A). Similarly, palmitate significantly increased apoptosis compared to control conditions. The apoptotic effect was approximately 50% greater at the higher concentration of palmitate (0.5mM), further and significantly increasing apoptosis (Fig 1B).

**Fig 2:**
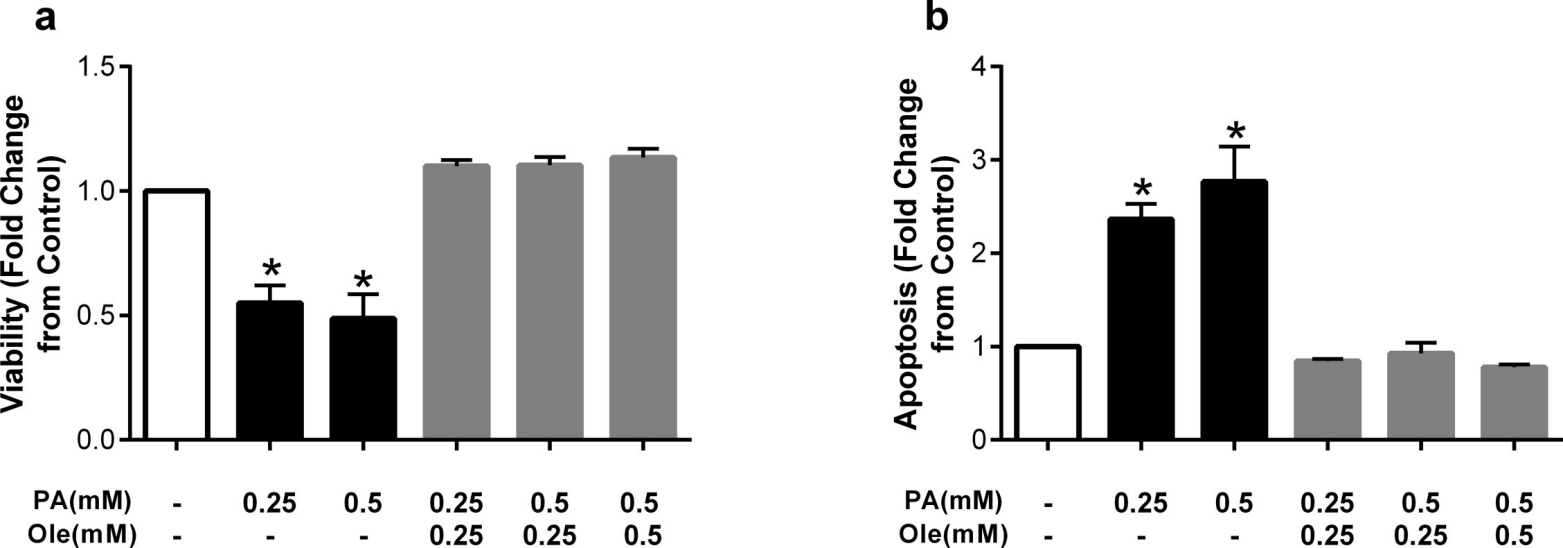
The monounsaturated fatty acid oleate prevents detrimental effects of palmitate. Fold change from control in a) viability and b) apoptosis after treatment with palmitate (PA) at 0.25 or 0.5mM, or co-treatment with the unsaturated fatty acid oleate (Ole). Data are expressed as mean±SEM; n = 4–5 independent experiments; *p<0.05 vs control.

We next examined whether addition of the monounsaturated fatty acid oleate (C18:1Δ9) altered palmitate-induced cell death. As shown in Fig 2A, 0.25 and 0.5mM of palmitate again induced significant reductions in cell viability, and co-incubation with matched concentrations of oleate completely reversed these reductions. Remarkably, at the high palmitate concentration (0.5mM), co-incubation with oleate at a 2:1 (palmitate:oleate) ratio (0.25mM) was also sufficient to restore cell viability. Similar results were found for apoptosis, such that co-incubating with oleate at a 1:1 or 2:1 (palmitate:oleate) ratio was sufficient to completely abrogate palmitate-induced cell apoptosis (Fig 2B).

**Fig 3:**
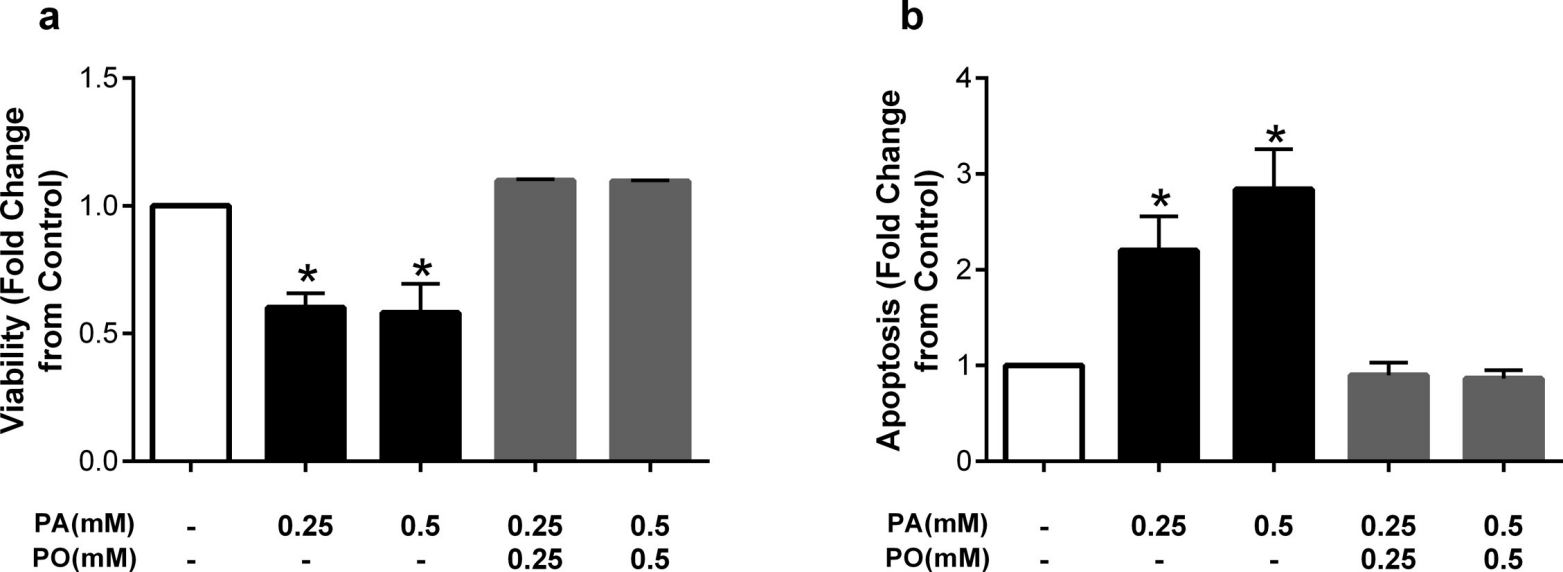
The monounsaturated fatty acid palmitoleate prevents detrimental effects of palmitate. Fold change from control in a) viability and b) apoptosis after treatment with palmitate (PA) at 0.25 or 0.5mM, or co-treatment with the unsaturated fatty acid palmitoleate (PO). Data are expressed as mean±SEM; n = 4–6 independent experiments; *p<0.05 vs control.

The protective effects of oleate on cell death and dysfunction have been demonstrated in other cell types, including hepatocytes and pancreatic β-cells [19] [21]. To examine whether these protective effects are specific to oleate or occur with other monounsaturated fatty acids, we next co-incubated HUVECs with palmitate and palmitoleate (C16:1Δ9). As with oleate, concentration-matched co-incubation with palmitoleate prevented the detrimental effects of palmitate on viability and apoptosis at both 0.25 and 0.5mM (Fig 3A and 3B), suggesting that the protective effects are not specific to oleate and may be a class effect of monounsaturated fatty acids.

**Fig 4:**
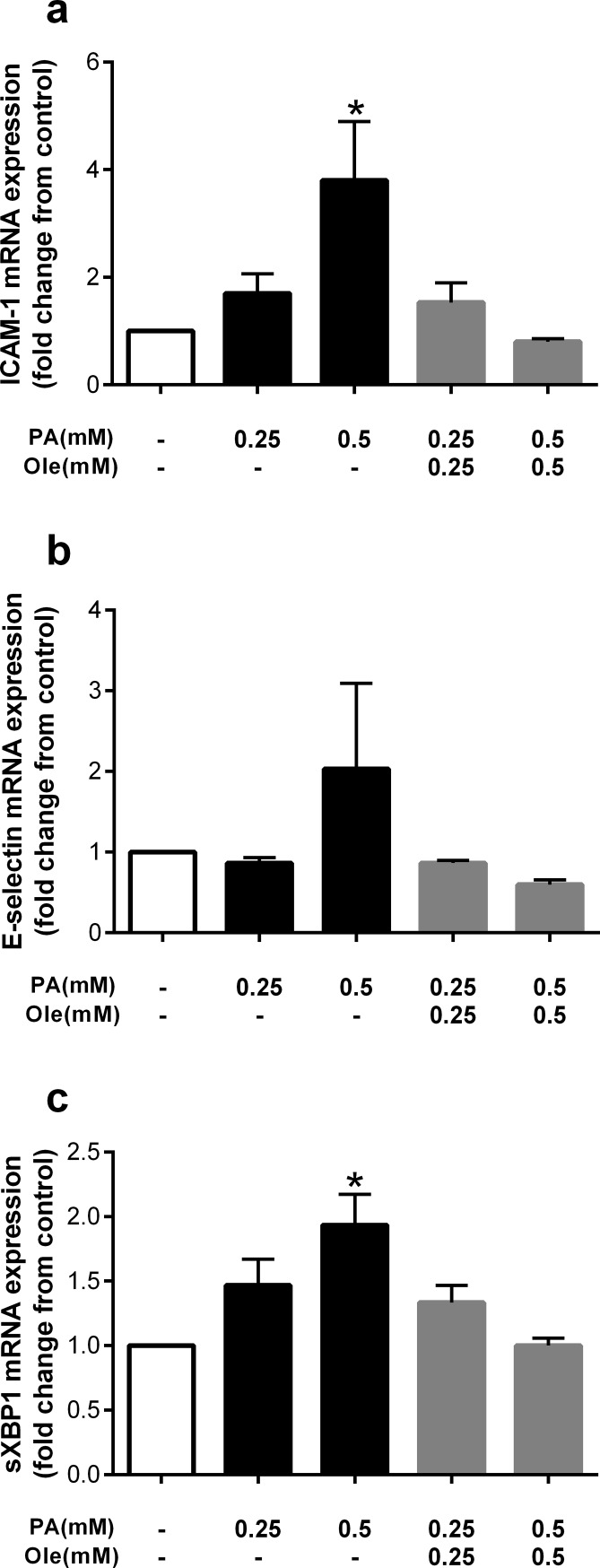
The monounsaturated fatty acid oleate prevents palmitate-induced markers of cellular adhesion and ER stress. Fold change from control in a) ICAM-1, b) E-selectin and c) sXBP1 expression after treatment with palmitate (PA) at 0.25 or 0.5mM, or co-treatment with the unsaturated fatty acid oleate (Ole). Data are expressed as mean±SEM, n = 3 independent experiments, *p<0.05 vs control.

Given the protective effects of these monounsaturated fatty acids against the detrimental effects of palmitate on viability and apoptosis, and to begin to explore the potential mechanisms mediating this effect, we measured gene expression of cellular adhesion and endoplasmic reticulum (ER) stress markers, both of which have been implicated in endothelial dysfunction [12]. Similar to the protective effects on viability and apoptosis, concentration-matched co-incubation with oleate (0.5mM) significantly attenuated expression of the cellular adhesion marker ICAM-1 (Fig 4A). Similarly, E-selectin expression tended to be increased with palmitate and attenuated with co-treatment with oleate at 0.5mM, although these changes were not statistically significant (Fig 4B). The ER stress-associated gene, sXBP1 was significantly increased with palmitate treatment and attenuated by co-treatment with oleate at 0.5mM (Fig 4C).

**Fig 5:**
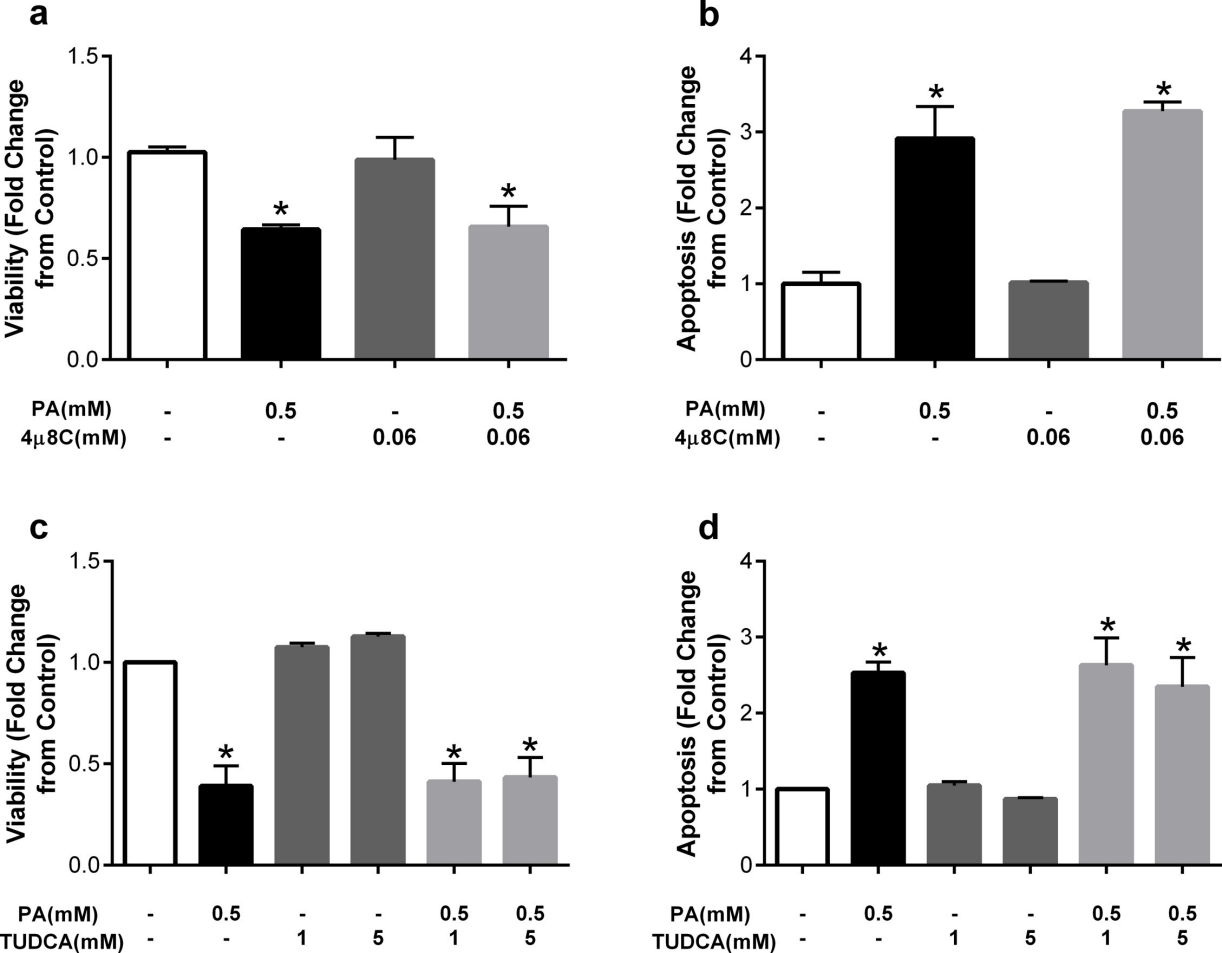
The small molecule IRE1 inhibitor 4μ8C or ER stress inhibitor TUDCA, do not prevent the detrimental effects of palmitate. Fold change from control in a) viability and b) apoptosis after treatment with palmitate (PA) at 0.5mM or co-treatment with the IRE1 inhibitor 4μ8C at 0.06mM. Fold change from control in a) viability and b) apoptosis after treatment with palmitate (PA) at 0.5mM or co-treatment with TUDCA at 1 and 5mM. Data are expressed as mean±SEM, n = 2–4 independent experiments, *p<0.05 vs control.

We next explored whether the increase in ER stress induced by palmitate was causal in palmitate-mediated cell death. To this end, we examined the potential protective effects of the general ER stress inhibitor TUDCA. In light of the significant increase in sXBP1, we also utilized 4μ8C, a small molecule inhibitor of IRE1, the upstream regulator of XBP1 splicing. Co-incubation of palmitate and 4μ8C did not prevent palmitate-induced decreases in viability and increases in apoptosis (Fig 5A and 5B). Similarly, co-incubation of palmitate and TUDCA at a low (1mM) or high (5mM) dose did not prevent the detrimental effects of palmitate (Fig 5C and 5D). These results suggest that although palmitate induces ER stress, this induction is not critical to palmitate-mediated cell death.

**Fig 6:**
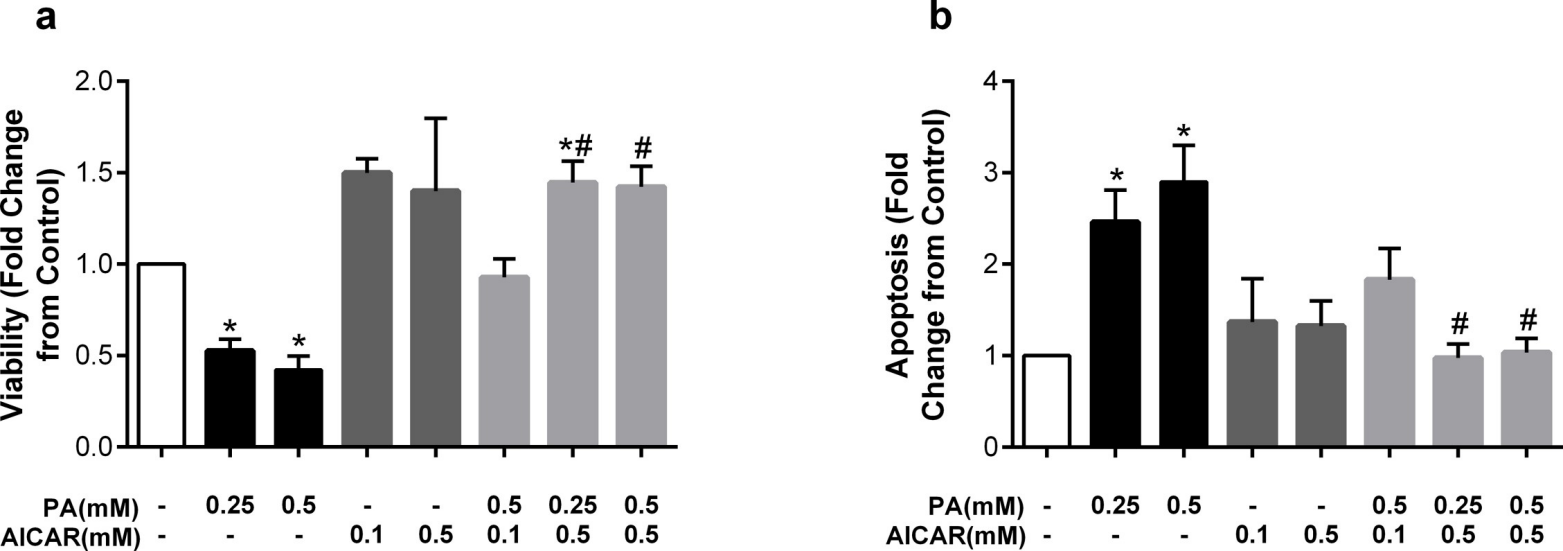
The AMPK activator AICAR prevents detrimental effects of palmitate. Cells were pretreated with AICAR (0.1 or 0.5mM) for 2hr then co-incubated with palmitate (PA). Fold change from control in a) viability and b) apoptosis after treatment with palmitate (PA), AICAR, or a combination of both. Data are expressed as mean±SEM; n = 2–6 independent experiments; *p<0.05 vs control, #p<0.05 vs both PA concentrations.

Having established that monounsaturated fatty acids are capable of reversing palmitate-induced lipoapoptosis and that ER stress is not required for palmitate-mediated lipoapoptosis, we sought to examine other mechanisms underlying this protection. AMP-activated protein kinase (AMPK) is a critical energy sensor that promotes beneficial effects on hyperlipidemia [24]. AMPK has also been shown to protect against saturated fatty acid-induced cell damage in a variety of cell types [25–27]. We therefore asked whether AMPK may be an important mechanism underlying the detrimental effects of palmitate and beneficial effects of oleate on endothelial cell viability and apoptosis. To do so, we examined the potential protective effects of AICAR, an established AMPK activator [28], on palmitate-mediated lipoapoptosis. 26hr incubation with AICAR alone at 0.1 and 0.5mM, in the absence of fatty acids, increased viability by approximately 50% above control levels (Fig 6A). AICAR (0.1 and 0.5mM) was then added to HUVECs 2hr prior to the addition of palmitate, then co-incubated for 24h. AICAR at 0.1mM completely prevented palmitate-mediated reductions in viability; whereas AICAR at 0.5mM further increased viability above control values even in the presence of high palmitate concentration (0.5mM) (Fig 6A). AICAR alone had no effect on apoptosis, but both high and low concentrations of AICAR were sufficient to prevent palmitate-induced apoptosis at both 0.25 and 0.5mM (Fig 6B). Collectively, these results indicate that the AMPK activator AICAR protects endothelial cells from the deleterious effects of palmitate-mediated cell death.

**Fig 7:**
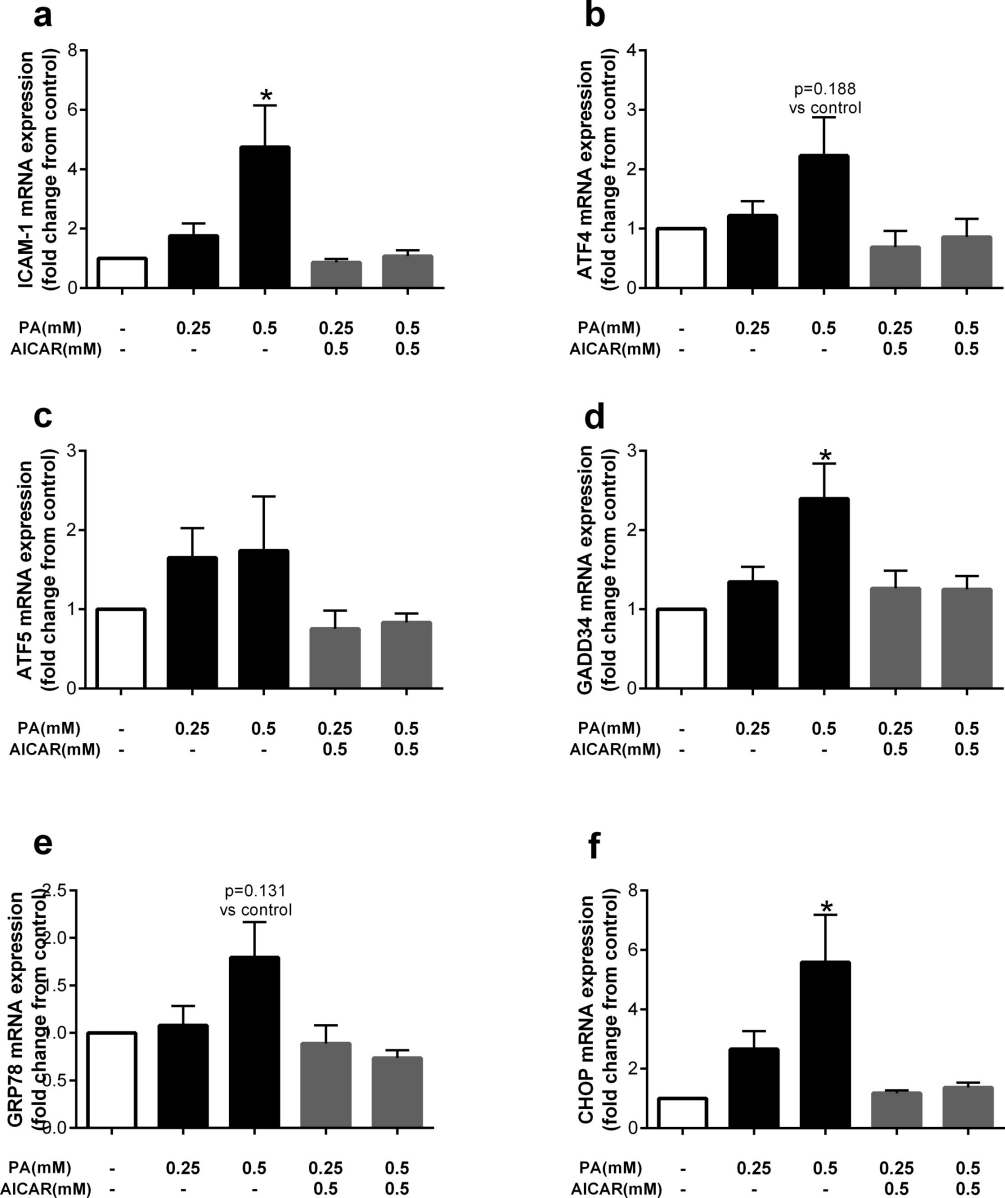
The AMPK activator AICAR attenuates palmitate-induced markers of cellular adhesion and ER stress. Cells were pretreated with AICAR (0.5mM) for 2hr then co-incubated with palmitate (PA). Fold change from control in a) ICAM-1, b) ATF4, c) ATF5, d) GADD34, e) GRP78 and f) CHOP expression after treatment with palmitate (PA) 0.25 and 0.5mM, or co-treatment with the AMPK activator AICAR (0.5mM). Data are expressed as mean±SEM, n = 3 independent experiments, *p<0.05 vs control.

Although ER stress inhibition with 4μ8C or TUDCA did not attenuate the detrimental effects of palmitate, it is possible that oleate and AICAR act via similar mechanisms to prevent palmitate-induced lipoapoptosis. Thus, we examined the effect of AICAR on cellular adhesion and ER stress-related genes given that oleate was found to attenuate the ER stress marker, sXBP-1. Similar to previous data, palmitate significantly increased expression of ICAM-1, which was attenuated by co-treatment with AICAR (Fig 7A). Consistent with the effect of oleate, five markers of ER stress, including ATF4, ATF5, GADD34, GRP78, and CHOP tended to be increased by palmitate and attenuated with AICAR treatment (Fig 7B–7F).

**Fig 8:**
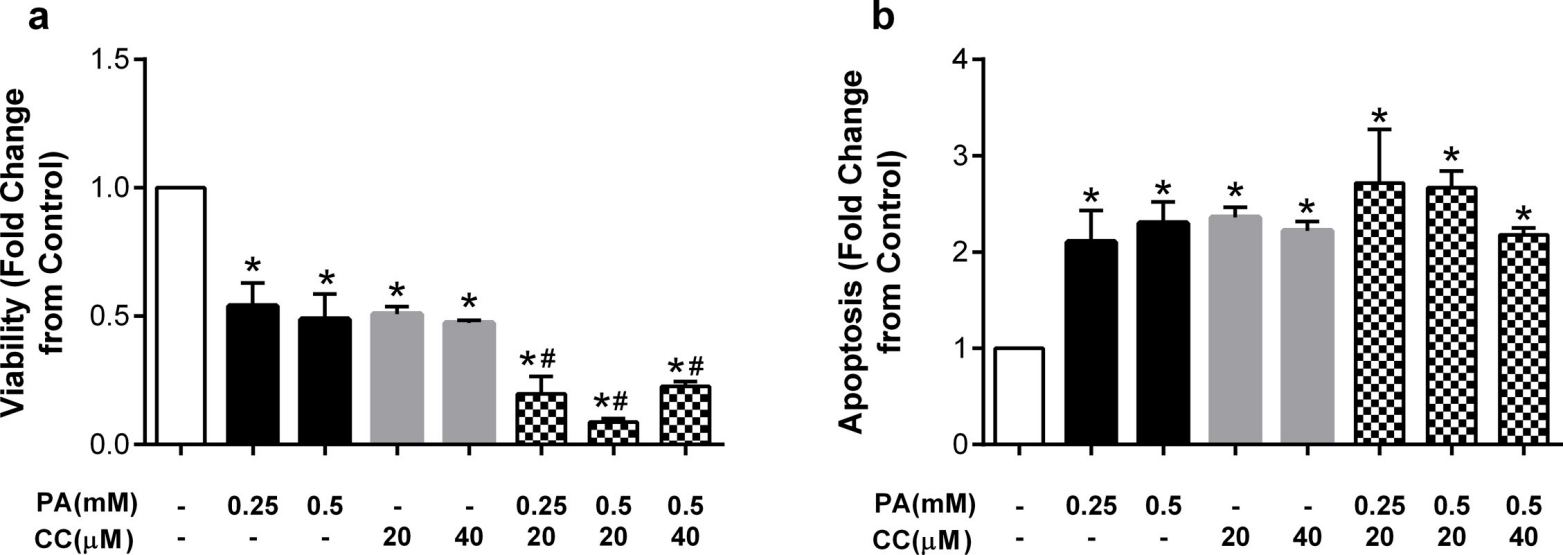
The AMPK inhibitor CC exacerbates palmitate-induced decreases in viability. Cells were pretreated with compound C (CC; 20 or 40μM) and then co-incubated with palmitate (PA). Fold change from control in a) viability and b) apoptosis after treatment with palmitate (PA), compound C (CC), or a combination of both. Data are expressed as mean±SEM; n = 3–5 independent experiments; *p<0.05 vs control, #p<0.05 vs both PA concentrations.

To provide further evidence of a role for AMPK, we next examined the modulatory effects of compound C (CC), a well-established AMPK inhibitor. Incubation of endothelial cells with compound C at 20 or 40μM in the absence of fatty acids reduced HUVEC viability to a similar magnitude as that observed with palmitate (Fig 8A). Compound C was then added to endothelial cells 1h prior to palmitate and co-incubated for the subsequent 24h. The addition of compound C significantly exacerbated the effects of palmitate such that the significant reduction in cell viability with palmitate alone was further reduced by approximately 30% (Fig 8A). Compound C did not exhibit dose-dependent effects. Incubation of HUVECs with compound C in the absence of palmitate also elicited increases in apoptosis (Fig 8B). However, unlike the effects on viability, compound C did not significantly exacerbate palmitate-induced increases in apoptosis (Fig 8B).

**Fig 9:**
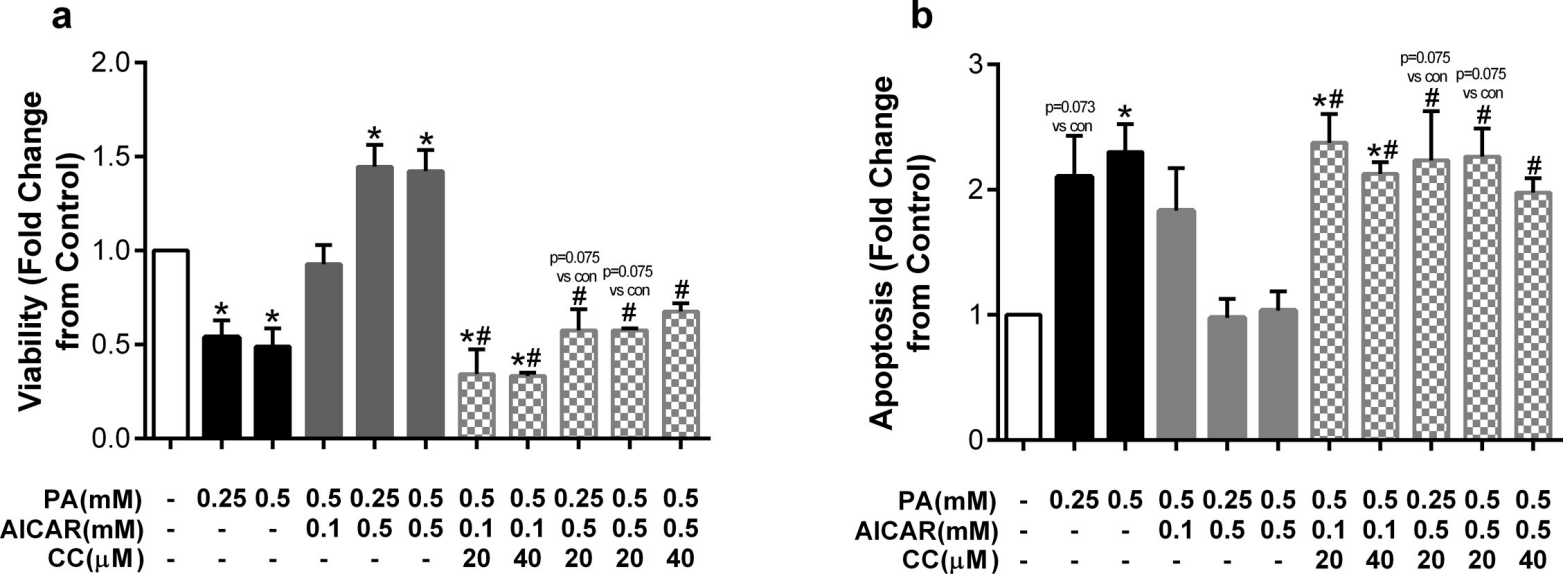
The AMPK inhibitor CC prevents protective effects of AICAR against palmitate. Cells were pretreated with AICAR (0.1 or 0.5mM) alone or in combination with compound C (CC; 20 or 40μM) and then co-incubated with palmitate (PA). Fold change from control in a) viability and b) apoptosis after treatment with palmitate (PA), compound C (CC), and AICAR. Data are expressed as mean±SEM; n = 3–6 independent experiments; *p<0.05 vs control, #p<0.05 vs PA+AICAR at equimolar doses.

Next, to confirm that compound C and AICAR were acting via the same mechanism (i.e. inhibiting and activating AMPK, respectively) we examined the effects of co-incubating both substances with palmitate. As expected, AICAR protected endothelial cells from reductions in viability and increases in apoptosis, and the addition of CC prevented these effects (Fig 9A and 9B), confirming the two modulators are acting via the same mechanism.

**Fig 10:**
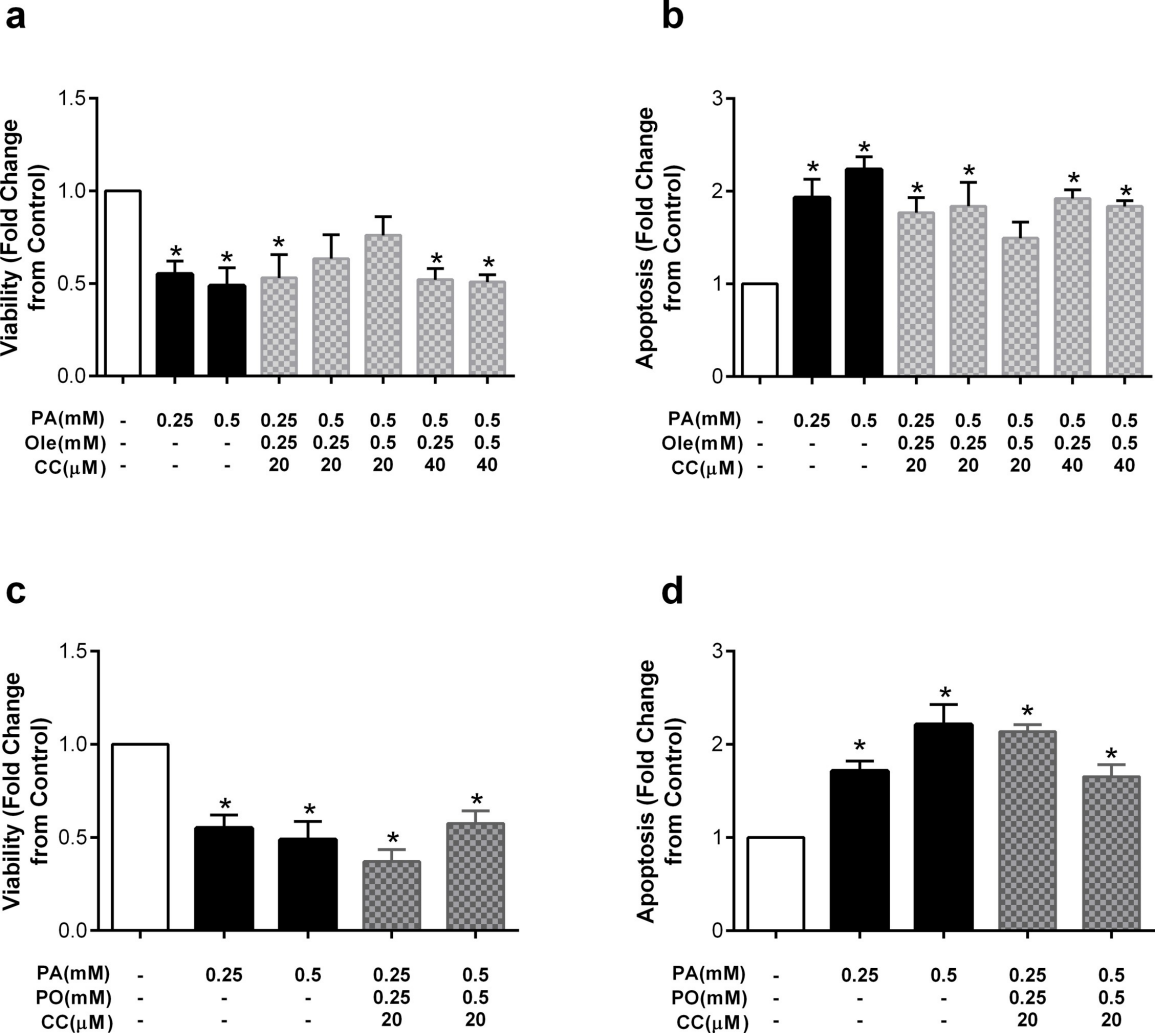
The AMPK inhibitor CC prevents protective effects of the monounsaturated fatty acids oleate and palmitoleate. Cells were pretreated with compound C (CC; 20 or 40μM) and then co-incubated with a combination of palmitate (PA) and oleate (Ole) or palmitoleate (PO). Fold change from control in a) viability with Ole, b) apoptosis with Ole, c) viability with PO and d) apoptosis with PO after co-treatment with palmitate (PA) and compound C (CC). Data are expressed as mean±SEM; n = 3–5 independent experiments; *p<0.05 vs control.

Lastly, to examine whether AMPK activation may be mediating the protective effects of the monounsaturated fatty acid oleate, we incubated HUVECs with a combination of palmitate, oleate and compound C. Compound C at both 20 and 40uM mitigated the effect of oleate on cell viability such that oleate was no longer protective (Fig 10A). The two exceptions were palmitate at 0.5mM and oleate at both 0.25 and 0.5mM in combination with compound C at 20uM; under these conditions, there was a ~25% decrease in viability compared to control, although the change did not reach statistical significance. Similarly, compound C also attenuated the protective effect of oleate on apoptosis (Fig 10B). Similar effects were observed for the protective effects of palmitoleate; that is, compound C completely abrogated the protective effects of palmitoleate on cell viability (Fig 10C) and attenuated the protective effects on apoptosis (Fig 10D).

## Discussion

The present study examined the effects of the saturated fatty acid palmitate on endothelial cell viability and apoptosis, and the potential protective effects of monounsaturated fatty acids. Our results show that incubation of HUVECs with palmitate reduced viability and increased apoptosis in a dose-dependent manner. Furthermore, co-incubation with two different monounsaturated fatty acids, oleate and palmitoleate, at a 1:1 or 2:1 ratio (saturated:unsaturated fatty acid) prevented the deleterious effects of palmitate. Oleate treatment also attenuated palmitate-induced cellular adhesion and ER stress markers, ICAM-1 and sXBP-1, respectively. Interestingly, chemical inhibition of ER stress did not prevent palmitate-induced lipoapoptosis. The effects of palmitate on viability and apoptosis were also prevented by the AMPK activator AICAR, and exacerbated by the AMPK inhibitor compound C. Converse to chemical ER stress inhibition, co-treatment with AICAR prevented palmitate-induced cellular adhesion and ER stress. Lastly, compound C negated the protective effects of oleate and palmitoleate. To the best of our knowledge, this is the first report to demonstrate that monounsaturated fatty acids can protect against palmitate-induced cell death in endothelial cells, and that both the deleterious effects of palmitate and the protective effects of oleate and palmitoleate are mediated, at least in part, via AMPK.

We chose to examine the detrimental effects of palmitate because it is the most abundant long chain saturated fatty acid in circulation, and both dietary and circulating levels have been associated with metabolic disease risk [17] [29] [30]. Clinical and cell-based studies have also linked palmitate to endothelial dysfunction [31] [32]. The ability of palmitate to reduce cell viability and induce apoptosis has been demonstrated in various cell types [33] [34], including bovine aortic endothelial cells [18]. Lee and colleagues demonstrated in HUVECs that palmitate at 0.5mM induced endothelial cell apoptosis and reduced viability. These effects were accompanied by an increase in reactive oxygen species and attenuated by the polyunsaturated fatty acid, eicosapentanoic acid [35]. Our results support and extend these findings by demonstrating that lower palmitate levels (i.e. 0.25mM) that are more commonly observed in free-living individuals can also induce endothelial cell death over a similar timeframe. Furthermore, our data extend these findings by providing evidence for monounsaturated fatty acids (i.e. oleate and palmitoleate) in their protective role against palmitate-induced lipoapoptosis.

Unlike saturated fatty acids, diets high in unsaturated fatty acids have been linked to reduced cardiovascular events and dysfunction [36]. The monounsaturated fatty acid oleate has garnered particular focus because of its presence in olive oil and importance to the Mediterranean diet [37, 38]. *In vitro* studies have supported human trials by demonstrating that unsaturated fatty acids are less toxic than long chain saturated fatty acids in various cell types, including endothelial cells [23, 35, 39, 40]. For example, Harvey et al. and Artwohl et al. demonstrated that endothelial cell dysfunction caused by the saturated fatty acid stearate (C18:0) was mitigated by co-incubation with oleate or eicosapentanoic acid [23, 39]. These studies coincide with work in other cell types, such as hepatocytes and pancreatic β-cells, in which oleate has been shown to mitigate saturated fatty acid cell toxicity [19, 20, 41]. In the present study we demonstrate that these protective effects are not unique to oleate, but also occur following co-incubation with palmitoleate, a sixteen carbon monounsaturated fatty acid formed by the action of stearoyl-CoA desaturase. Although some population studies have suggested a positive relation between palmitoleate and metabolic disease [42, 43], more recent studies have identified palmitoleate as a lipokine with protective effects in a variety of tissues [44, 45]. Cimen et al., recently reported that palmitoleate treatment prevented atherosclerotic plaque formation and vascular inflammation in apolipoprotein E-deficient mice [46]. In cell culture models, palmitoleate attenuated palmitate-induced apoptosis in hepatocytes, and palmitate, but not palmitoleate, caused insulin resistance in endothelial cells [47]. The present study is the first to demonstrate that palmitoleate is protective against palmitate-induced cell death in endothelial cells. It will be interesting to determine in future studies if these results portend a protective effect of palmitoleate in clinical endothelial dysfunction.

Both endothelial cell activation and ER stress have been implicated in the development of metabolic diseases, although the latter’s role in endothelial dysfunction is less established. We found that palmitate treatment induced the cellular adhesion molecule ICAM-1 and several ER stress-related markers, which is supported by prior findings [22, 48]. Previous data has also supported the attenuation of cell adhesion molecules and ER stress with improved endothelial function *in vitro* and *in vivo* [15, 48, 49]. Given this link, we examined the effect of palmitate on HUVEC cell adhesion and found that palmitate-induced ICAM-1 expression was attenuated by co-treatment with the monounsaturated fatty acid, oleate. Similarly, the palmitate-induced ER stress marker, sXBP-1 was attenuated by co-treatment with oleate. Prior studies have also found saturated fatty acids to induce endothelial cell activation and ER stress [22, 48]. Our data corroborate and extend these findings by demonstrating a role of monounsaturated fatty acids in their protection against the deleterious effects of palmitate.

The metabolic sensing kinase, AMPK, coordinates diverse anabolic and catabolic processes and controls metabolic health [50]. Previous *in vitro* and *in vivo* studies have shown that activation of AMPK ameliorates endothelial dysfunction [48]. The protective effects in endothelial cells has been attributed to increases in p-eNOS [48] and decreases in ROS production [18]. Palmitate was shown to inhibit phosphorylation of AMPK in endothelial cells via a ceramide-dependent PP2A activation [51] and activation of AMPK was shown to alleviate ER stress [48]. Along with the above evidence, our data utilizing an activator and inhibitor of AMPK suggests that alterations in AMPK activation may underlie the deleterious effects of palmitate and protective effects of monounsaturated fatty acids in endothelial cells.

Several limitations of the current study should be noted. For example, the *in vitro* nature of the study is several steps removed from a free-living condition, and thus caution should be taken before inferring clinical implications of the findings. In this regard, the reductionist approach of adding individual nutrients to cell culture models is helpful in examining the cellular effects of those nutrients, but it is highly unlikely cells *in vivo* are exposed to individual nutrients in isolation. In an attempt to increase the external validity of the experiments, the concentrations of palmitate used are within the range of circulating levels in humans. In conclusion, we found that the saturated fatty acid palmitate is a potent inducer of cell death, endothelial cell activation and ER stress in HUVECs, and that the monounsaturated fatty acids, oleate or palmitolate confer protection from the cytotoxic effects of palmitate in an AMPK-dependent manner. Direct chemical activation and inactivation of AMPK also mitigated and exacerbated, respectively, palmitate induced toxicity. In future studies, it will be important to corroborate these results in animal and human studies that examine the effects of replacing dietary palmitate with oleate or palmitoleate on endothelial dysfunction; and to explore the vascular effects of activating AMPK *in vivo*.

## Supporting information

S1 Data Data underlying Figs 1–10. (XLSX)
